# Operational outcomes of propofol sedation versus fentanyl, midazolam and diphenhydramine sedation for endoscopies and colonoscopies at an academic medical center

**DOI:** 10.1371/journal.pone.0294418

**Published:** 2023-11-27

**Authors:** Andrew L. Mariotti, Jack Pattee, Steven A. Edmundowicz, Terran D. Hardesty, Savita M. Sharma, M. G. Lemley, Scott D. Rist, Nathaen Weitzel, Adeel A. Faruki

**Affiliations:** 1 School of Medicine, University of Colorado, Aurora, Colorado, United States of America; 2 Colorado School of Public Health, University of Colorado, Aurora, Colorado, United States of America; 3 Department of Gastroenterology, University of Colorado School of Medicine, Aurora, Colorado, United States of America; 4 Endoscopy Lab, UCHealth, Aurora, Colorado, United States of America; 5 Department of Anesthesiology, University of Colorado School of Medicine, Aurora, Colorado, United States of America; 6 Department of Anesthesiology, MD Anderson Cancer Center, Houston, Texas, United States of America; IRCCS: IRCCS Ospedale San Raffaele, ITALY

## Abstract

**Background:**

On July 1^st^, 2021, the University of Colorado Hospital (UCH) implemented new sedation protocols in the luminal gastrointestinal (GI) suite. GI proceduralist supervised, Nurse Administered Sedation with fentanyl, midazolam, and diphenhydramine (NAS) sedation was transitioned to Monitored Anesthesia Care with propofol under physician anesthesiologist supervision (MAC).

**Objective:**

To determine if there are statistically significant reductions in Sedation-Start to Scope-In time (SSSI) when using Monitored Anesthesia Care with propofol (MAC) versus Nurse Administered Sedation with fentanyl, midazolam, and diphenhydramine (NAS). Secondary objectives were to determine if statistically significant improvements to other operational times, quality measures, and satisfaction metrics were present.

**Method:**

This study was a retrospective analysis of a natural experiment resultant of a change from NAS to MAC sedation protocols. Outcomes for NAS protocols from 1/1/21–6/30/21 were compared to outcomes of MAC protocols from the dates 8/1/21–10/31/21. Results were analyzed using Quasi-Poisson regression analysis and stratified based on upper GI, lower GI, and combined procedures. Patient demographic data including age, biological sex, comorbidities, and BMI, were adjusted for in the analysis. ASA matching was not performed as nursing sedation does not use ASA classifications. Pre-anesthesia co-morbidities were assessed via evaluation of a strict set of comorbidities abstracted from the electronic medical record. Perioperative operational outcomes include Sedation Start to Scope-In (SSSI), In-Room to Scope-In Time (IRSI), Scope Out to Out of Room (SOOR), Total Case Length (TCL), and Post Anesthesia Care Unit Length of Stay (PACU LOS). Quality outcomes include PACU Administered Medications (PAM), and Clinician Satisfaction Scores (CSS).

**Results:**

A total of 5,582 gastrointestinal (GI) endoscopic cases (upper, lower, and combined endoscopies) were observed. Statistically significant decreases in SSSI of 2.5, 2.1, and 2.2 minutes for upper, lower, and dual GI procedures were observed when using MAC protocols. A statistically significant increase in satisfaction scores of 47.0 and 19.6 points were observed for nurses and proceduralists, respectively, when using MAC.

**Conclusion:**

MAC protocols for endoscopic GI procedures at UCH led to statistically significant decreases in the time required to complete procedures thus increasing operational efficiency.

## Introduction

The use of sedation in gastroenterology (GI) for endoscopic procedures has been employed for decades. Despite this, best practices for sedation during endoscopic GI procedures—especially as they pertain to operational flow—are not well established.

Fentanyl, midazolam [1, 2], meperidine [3], and sufentanil [4] exemplify popular benzodiazepine and opioid choices for sedation during endoscopic procedures. Research on depth of sedation, recovery time, complications, rate of administration [5], and patient satisfaction [6] have shown these pharmaceuticals to be safe and effective. Over the ensuing years, the use of propofol has risen due to superior clinical effectiveness [7]. While propofol provides reliable sedation with fewer side-effects, questions remain as to whether its use is necessary for endoscopic procedures [8]. Further, operational outcomes comparisons between propofol, and benzodiazepine and opiate combinations are poorly described, leaving best practices for sedation during endoscopic GI procedures unresolved [9, 10]. Several analyses investigating sedation effectiveness based on type of administering clinician have been performed. Comparisons of effectiveness when sedation is administered by licensed physicians, advanced practice providers [11], or registered nurses (RN) have been explored, though results are inconclusive. In certain cases, RN sedation with propofol was found to be safer than physician administered sedation [12, 13], while other studies cite higher rates of serious complications [14]. Like the debate over sedatives, definitive evidence for which type of clinician provides the greatest operational efficiency is inconclusive.

With increasing emphasis on value-based care [15, 16] and larger populations requiring endoscopic procedures [17], the importance of identifying best sedation practices for endoscopic procedures continues to grow. Optimal process flow is yet another area of endoscopic GI sedation lacking definitive best practices [18].

In this study, researchers compared the operational outcomes of Nurse Administered Sedation using fentanyl, midazolam, and diphenhydramine under the supervision of a GI proceduralist (NAS), to Monitored Anesthesia Care using propofol administered by an advanced practice provider under physician anesthesiologist supervision (MAC). As MAC can range widely in terms of depth of anesthetic, for the purposes of this study, MAC is used as the term to describe the type of anesthesia performed by the anesthesia care team. A comparison of the operational, quality, and satisfaction outcomes between these techniques are evaluated to better understand the best practices for sedative type, administering clinician type, and process flow. This manuscript provides a unique addition to currently available literature regarding best practices for sedation during endoscopic GI procedures at a large academic medical center. We hypothesized that the use of Monitored Anesthesia Care improves primary and secondary operational and quality outcomes as compared to Nurse Administered Sedation.

## Materials & methods

On July 1^st^, 2021, the University of Colorado Hospital (UCH)—a quaternary care, academic medical center—implemented new sedation procedures in the endoscopic GI procedural room, shifting from NAS to MAC sedation protocols. Colorado Multiple Institution Review Board Exemption #21–4995 was obtained on January 13^th^, 2022, by the principal investigator, and data was observed retrospectively as a single cohort study using Strengthening the Reporting of Observational studies in Epidemiology (STROBE) guidelines.

**Fig 1:**
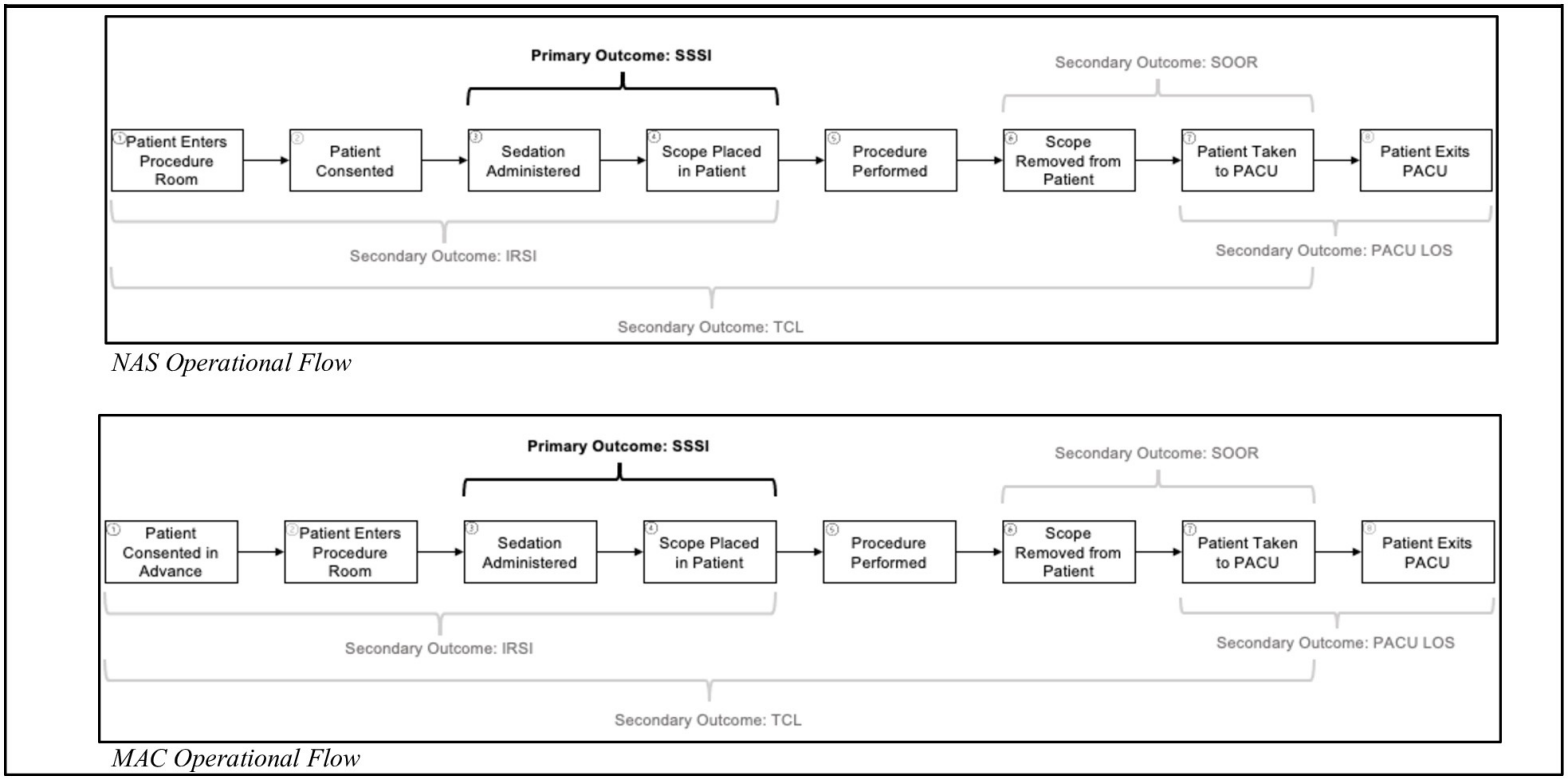
Process flow map for both NAS and MAC procedures indicating the steps in the endoscopy process. Start and end points for primary and secondary outcomes are noted in brackets.

The difference in sedation methods between NAS and MAC are comprised of three distinct aspects which include type of administering clinician, type of sedative, and patient flow during endoscopic GI procedures. For type of administering clinician, NAS protocols employ the use of sedation administration trained registered nurses (RNs) to administer sedatives under the supervision of a certified GI proceduralist. RNs administering sedative must also perform the duties of a circulating RN in combination with their sedation responsibilities. MAC protocols employ the use of Certified Anesthesia Assistants (CAA), Certified Registered Nurse Anesthetists (CRNA) under the supervision of a physician anesthesiologist to administer sedative agents as their sole responsibility. The coverage model primarily utilized at the UCH GI procedural unit for the anesthesia care team model includes one physician anesthesiologist and three to four CAA or CRNAs. For type of sedative administered, NAS protocols use intravenous forms of synthetic opioid fentanyl, the benzodiazepine midazolam, and the antihistamine diphenhydramine to achieve moderate sedation colloquially known as “conscious sedation.” In contrast, MAC protocols employ the sole use of propofol, a non-barbiturate sedative, to achieve the appropriate depth of sedation for the procedure without placement of an airway. During implementation of MAC, a notable difference was made to patient flow by changing the time when informed consent was performed during the overall process. During the NAS protocols, consenting occurred in the procedure room but after transitioning to MAC protocols, consenting for both the procedure and anesthesia was performed in the preoperative holding area. A comparison of patient flow for NAS protocols and MAC protocols can be seen in Fig 1. Neither the type of administering clinician nor the class of sedative affected the order of steps in the process flow.

The results of this study were stratified based on three distinct endoscopic procedure types: upper GI, lower GI, and dual GI procedures. Upper GI procedures were performed via endoscope placement in the oropharynx to view the esophagus, stomach, and proximal small intestine. Lower GI procedures were performed via endoscope placement in the anus to view the rectum, sigmoid, descending, transverse, and ascending colons, and the cecum. Dual procedures were those in which both upper and lower GI procedures were performed sequentially on the same patient. These procedures required stratification to avoid confounding based on different risks, indications, and durations associated with each.

Operational outcomes for all patients over the age of eighteen who underwent sedation and endoscopic GI procedures at UCH with NAS protocols from 1/1/21–6/30/21 were compared to the outcomes of MAC protocols from the dates 8/1/21–10/31/21. Patients undergoing advanced GI procedures such as endoscopic retrograde cholangiopancreatography, endoscopic ultrasound, pill endoscopies, or those requiring general anesthesia with an endotracheal tube excluded from the data as they employ different sedation methods. Data from the month of July (7/1/21–7/31/21) was excluded to allow for a transitional period and to avoid skewed outcomes during the change from NAS to MAC protocols. ASA classification was not used to assess patients for nurse administered sedation and therefore was not documented for NAS cases. To better assess and correlate comorbid and physical status between cohorts due to a lack of ASA classification documentation with NAS protocol patients, researchers identified a set of relevant covariates from the MAC population that correlated to NAS populations. Pre-anesthesia co-morbidities were instead assessed via evaluation of a strict set of comorbidities that were de-identified and abstracted from the UCH electronic medical record (EMR) for those matching inclusion criteria. Outliers secondary to inaccurate reporting of data in the UCH EMR were removed from this study.

Relevant covariates included age, genetic sex, body mass index (BMI), a set of pertinent comorbidities (apnea, asthma, coronary artery disease, gastroesophageal reflux disease (GERD), hepatic dysfunction, hypertension, kidney disease, liver disease, pulmonary hypertension, valvular disease), and procedure type (upper GI, lower GI, and dual procedures). The demographics of both cohorts can be seen in Table 1.

**Table 1:** Distribution of clinical covariates in the study population, stratified by sedation type.

	MAC	NAS	Overall
	(N = 1094)	(N = 4758)	(N = 5852)
**Sex**			
Female	587 (53.7%)	2,533 (53.2%)	3,120 (53.3%)
Male	507 (46.3%)	2,225 (46.8%)	2,732 (46.7%)
**Age**			
Mean (SD)	55.9 (15.1)	55.4 (14.7)	55.5 (14.8)
Median [Min, Max]	58.0 [19.0, 94.0]	57.0 [18.0, 93.0]	57.0 [18.0, 94.0]
**BMI**			
Mean (SD)	27.1 (5.62)	27.0 (5.18)	27.0 (5.27)
Median [Min, Max]	26.4 [14.2, 50.5]	26.2 [14.6, 51.3]	26.3 [14.2, 51.3]
**Procedure Type**			
Dual	105 (9.6%)	473 (9.9%)	578 (9.9%)
Lower GI	742 (67.8%)	3,087 (64.9%)	3,829 (65.4%)
Upper GI	247 (22.6%)	1,198 (25.2%)	1,445 (24.7%)
**Comorbidity Count**			
Mean (SD)	1.27 (1.26)	1.21 (1.29)	1.22 (1.29)
Median [Min, Max]	1.00 [0, 7.00]	1.00 [0, 7.00]	1.00 [0, 7.00]

The primary outcome, Sedation-Start to Scope-In time (SSSI), was chosen due to its operational significance and ability to measure the effect that type of administering clinician and sedative administered—defining features of MAC and NAS protocols—had on length of case. While many factors contribute to case length, SSSI is a direct representation of the factors influenced by the anesthesia team. Similarly, secondary outcomes of Scope-Out to Out-of-Room time (SOOR), Total Case Length (TCL), and Post Anesthesia Care Unit Length of Stay (PACU LOS) are operational outcomes representative of the change from NAS to MAC sedation protocols, chosen to assess operational efficiency more wholistically.

While the secondary outcome, In-Room to Scope-In time (IRSI), represents changes in administering clinician and sedative type, it most strongly reflects the change in process flow implemented during the transition to MAC sedation (Fig 1). Thus, IRSI times provided data that allowed the team to determine the effects of process flow changes. All primary and secondary outcome measures are clearly defined in Table 2.

**Table 2:** Primary and secondary outcome definitions.

**Primary Outcome, Operational**	
**Outcome**	**Definition**
Sedation Start to Scope-In (SSSI)	Time sedation begins to time scope is placed
**Secondary Outcomes, Operational**	
**Outcome**	**Definition**
In-Room to Scope-In Time (IRSI)	Time patient enters procedural room to time scope is placed
Scope Out to Out of Room (SOOR)	Time sedation ends to time patient exits procedural room
Total Case Length (TCL)	Time patient enters procedural room to time patient exits room
Post Anesthesia Care Unit Length of Stay (PACU LOS)	Time Patient enters PACU to time patient exits PACU
**Secondary Outcomes, Quality**	
**Outcome**	**Definition**
PACU Administered Medications (PAM)	Rate of pain medication administration in the PACU
Clinician Satisfaction Score (CSS)	Nurse and proceduralist satisfaction score for NAS or MAC protocols

Additionally, pain and nausea medications dosed in the PACU were analyzed for each patient in each cohort. Nurse and proceduralist satisfaction scores were also analyzed via a survey of 48 clinicians who took part in both MAC and NAS procedures and were asked to rate their satisfaction for each process respectively on a scale of 0–100. Satisfaction survey data were collected during March and April 2022, allowing clinicians to reflect on their experience with both protocols. All data is managed using REDCap electronic data tools hosted at the University of Colorado School of Medicine [19, 20].

Of the 6,008 subjects assessed for eligibility, 5,852 subjects satisfied inclusion criteria and were included in the analysis. To achieve an unbiased estimate of the difference in effects between sedation protocols, potential confounding factors were identified and accounted for. In this study, a confounder was any variable associated with both the ‘exposure’ (whether NAS or MAC sedation was administered) and the primary and secondary operational outcomes. Subjects were excluded if one or more of the following exclusion criteria was met: missing BMI (72 subjects), BMI greater than 100 (2 subjects) or less than 10 (1 subject), IRSI time greater than 120 minutes (9 subjects), SSSI time greater than 120 minutes (54 subjects), SOOR time greater than 120 minutes (11 subjects), TCL greater than 360 minutes (3 subjects), and PACU LOS greater than 360 minutes (24 subjects). The analytic dataset was comprised of 4,758 NAS cases and 1,094 MAC cases. In total, there were 1,445 upper GI, 3,829 lower GI, and 578 dual GI cases (Table 1).

The number of NAS cases evaluated in this study were much greater than the number of MAC cases. At the time of data collection, the MAC protocol case volumes were increasing slowly with the new process. The three months’ worth of MAC data represents the MAC ramp-up period. Due to the discrepancy in sample sizes, a power calculation was performed to ensure the sample sizes were both well powered. The power calculation was performed prior to data collection with a conservative assumption that 200 subjects would accrue per month. Given a three-month period for MAC and a six-month period for NAS, an expected sample size of 1,200 NAS cases and 400 MAC cases was reached. Using a two-sample two-sided t-test with a type I error rate of 0.05, a power at 80% was achieved to detect a sample size with Cohen’s D of 0.14, which is consistent with a ‘small’ effect. Given the observed sample was substantially larger than the conservatively assumed size, the study was well powered to detect a difference in the endpoints between NAS cases and MAC cases despite the disparity in their N-values.

The study design approximates a ‘natural experiment’, whereby the intervention (the transition from NAS to MAC protocols) was applied to a population. There is no known a priori reason why the population of patients during the NAS period would differ from the population of patients during the MAC period. We assessed whether the following potential confounders were balanced between the NAS and the MAC populations: age, genetic sex, body mass index (BMI), apnea, asthma, coronary artery disease, GERD, hepatic dysfunction, hypertension, kidney disease, liver disease, pulmonary hypertension, and valvular disease. The criteria of Zhang et al. [19] was applied to determine covariate balance; a covariate was determined to be balanced if the magnitude of the standardized mean difference was less than 0.1 and the variance ratio was less than 2. Note that variance ratio is applicable only to continuous covariates, i.e., age and BMI. Balance was assessed separately for upper, lower, and dual procedures.

It was determined that the following four baseline study variables were imbalanced between the NAS and MAC populations: asthma, liver disease, coronary artery disease, and GERD. To account for the potential confounding effects of these variables, a multiple generalized linear regression model was estimated with these four variables included as covariates alongside the respective treatment group. Given the observed skew and overdispersion in the distribution of the five outcome variables, endpoints were modeled via quasi-Poisson regression. The Poisson regression framework provided accurate estimation of whether the NAS or MAC protocol changed the rate at which patients progress through a given procedural stage, corresponding to the given outcome. This enabled testing for differences between NAS and MAC protocol subjects on the *multiplicative* scale. Given the right-skewed distribution of all five operational outcomes, identifying a difference on the multiplicative scale was likely more informative than identifying a mean difference (i.e., a difference on the *additive* scale), as estimation of the mean is sensitive to skew and outliers. The quasi-Poisson modeling allowed an estimation of standard errors robust to overdispersion, which was observed to some degree for all five outcomes. Unique quasi-Poisson models were estimated for each pairwise combination of procedure type and each operational outcome, entailing fifteen unique models.

Fisher’s exact test was used to analyze whether the administration rate of nausea and pain medications in the post-anesthesia care unit differed by sedation type following GI procedures. Satisfaction scoring was done via administration of a survey through REDCap for all clinicians who were involved with both NAS and MAC protocols. Respondents were asked to identify themselves as a nurse (N = 32) or proceduralist (MD or DO, N = 16) and self-report their level of satisfaction with each process based on a Likert scale ranging from one to one hundred. This was administered in March and April of 2022. Self-reported clinician satisfaction ratings on a Likert scale were chosen given its proven use in other studies to elicit accurate responses from clinicians [20, 21]. Results were compared separately for nurses and proceduralists. A two-sample t-test was used to analyze secondary satisfaction score outcomes—i.e., differences in nurse and physician satisfaction with NAS versus MAC.

## Results

**Fig 2:**
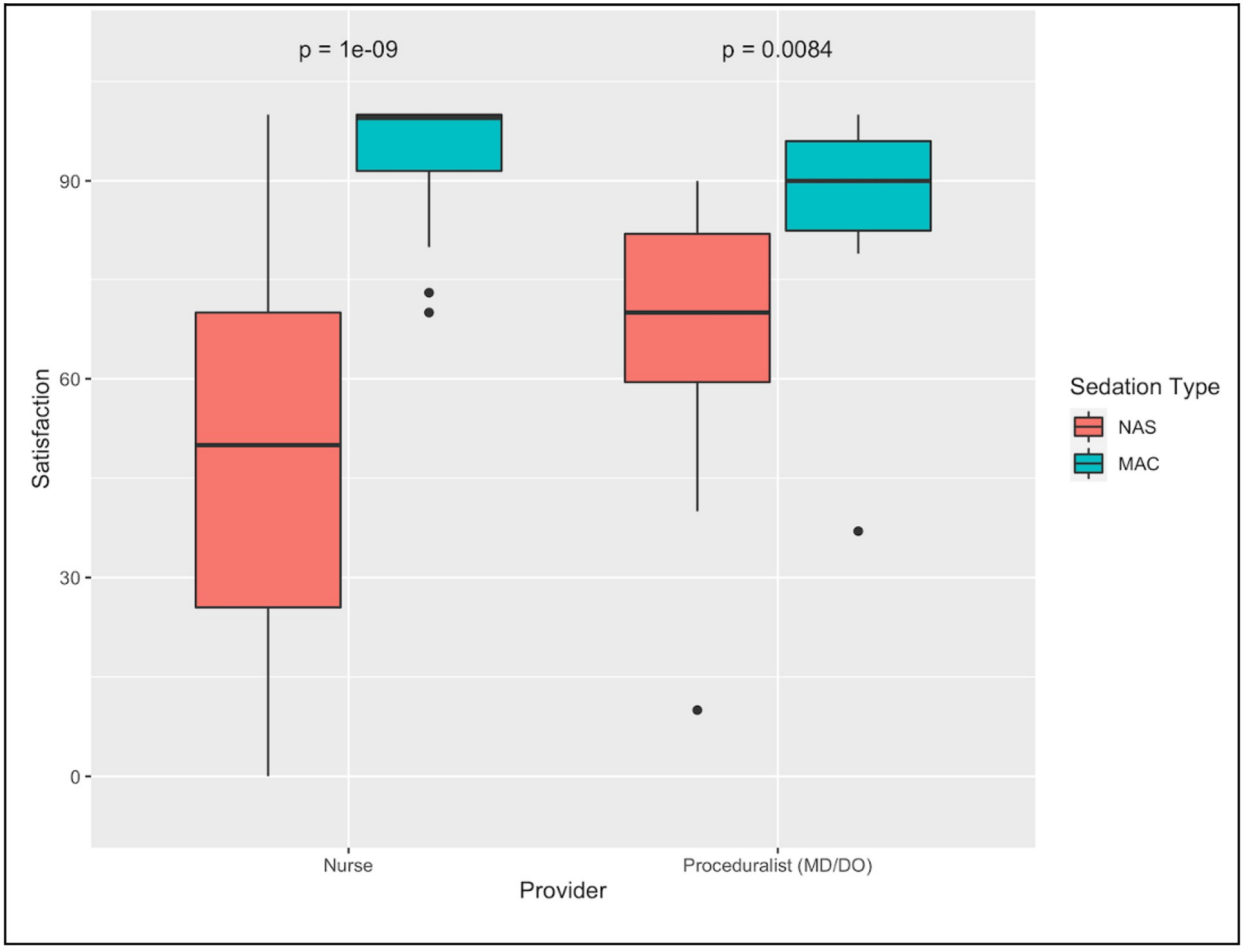
Love plots assessing the baseline covariate balance for upper, lower, and dual GI procedures. Vertical dotted lines denote the +/- 0.1 SMD cutoff.

Covariate balance was assessed uniquely for upper GI, lower GI, and dual GI procedures. For upper GI procedures, asthma was more common in MAC cases (SMD = -0.23) while liver disease was more common in NAS cases (SMD = 0.20). For lower GI procedures, GERD was more common in MAC cases (SMD = -0.11). For dual procedures, asthma (SMD = -0.12), coronary artery disease (SMD = -0.12), and GERD (SMD = -0.26) were more common in MAC cases. These four covariates (liver disease, GERD, asthma, and coronary artery disease) were thus included in the subsequent quasi-Poisson modeling to account for any potential confounding. The remaining nine potential confounders were not imbalanced for any of the three procedure types, and thus were not included in the subsequent model. A full accounting of covariate balance in all three procedure classes is described in Fig 2.

MAC’s effect on primary and secondary outcomes versus NAS as baseline, along with 95% confidence intervals are described in Table 3. For the primary outcome SSSI, MAC is associated with statistically significant reductions in average time. Reductions of 2.5, 2.1, and 2.2 minutes for upper, lower, and dual GI procedures were observed under MAC protocols as compared to NAS. Statistically significant differences at a type I error rate of 5% were observed whereby NAS is associated with a longer time to completion on average for IRSI, TCL and PACU LOS secondary operational outcomes in upper GI procedures. IRSI and TCL operational outcomes were statistically longer for lower GI procedures performed under NAS protocol. For combined procedures, a statistically significant reductions to IRSI and TCL, but not SOOR or PACU LOS time were observed.

**Table 3:** Operational time outcomes for upper, lower, and combined gastroenterology procedures.

Upper GI								
Outcome	MAC			NAS			P-value	Mean Difference (MAC–NAS)
	Mean Time	Lower 95% CI	Upper 95% CI	Mean Time	Lower 95% CI	Upper 95% CI		
IRSI	10.2	9.2	11.4	31.7	30.8	32.6	< 2.00 x 10^−16^	21.5
SSSI	7.5	7.2	7.9	10.0	9.8	10.2	< 2.00 x 10^−16^	2.5
SOOR	3.9	3.3	4.6	4.9	4.6	5.2	0.054	1.0
TCL	22.6	21.1	24.2	43.9	42.9	44.8	< 2.00 x 10^−16^	21.2
PACU LOS	40.0	36.8	43.4	46.9	45.3	48.6	0.0022	6.9
Lower GI								
Outcome	MAC			NAS			P-value	Mean Difference (MAC–NAS)
	Mean Time	Lower 95% CI	Upper 95% CI	Mean Time	Lower 95% CI	Upper 95% CI		
IRSI	9.9	9.3	10.6	29.1	28.6	29.7	< 2.00 x 10^−16^	19.2
SSSI	7.3	7.1	7.5	9.4	9.3	9.6	< 2.00 x 10^−16^	2.1
SOOR	3.5	3.2	3.9	4.2	4.0	4.4	0.019542	0.63
TCL	34.1	33.1	35.2	54.2	53.6	54.9	< 2.00 x 10^−16^	20.1
PACU LOS	37.7	36.2	39.3	39.7	39.0	40.5	0.139142	2.0
Dual GI								
Outcome	MAC			NAS			P-value	Mean Difference (MAC–NAS)
	Mean Time	Lower 95% CI	Upper 95% CI	Mean Time	Lower 95% CI	Upper 95% CI		
IRSI	10.0	8.3	11.9	31.8	30.3	33.3	< 2.22 x10^-16^	21.8
SSSI	8.0	7.3	8.7	10.2	9.8	10.5	< 4.42 x10^-6^	2.2
SOOR	4.1	3.4	5.1	4.9	4.5	5.4	0.63	0.75
TCL	48.0	44.9	51.3	69.3	67.5	71.1	< 2.22 x10^-16^	21.3
PACU LOS	46.0	40.6	52.1	49.7	46.9	52.5	1.00	3.7

Using Fisher’s exact test, a statistically significant difference in the prescribing rate of pain medication was observed between MAC and NAS (odds ratio 0.0, 95% CI 0.0–3.2, p = 3.9x10^-5^). This reflects the frequency with which patients required pain medication in the PACU and is abbreviated as PAM (Table 2). A significant difference was not observed in the prescribing rate of nausea medication (odds ratio 0.58, 95% CI 0.26–1.12, p = 0.12).

**Fig 3:**
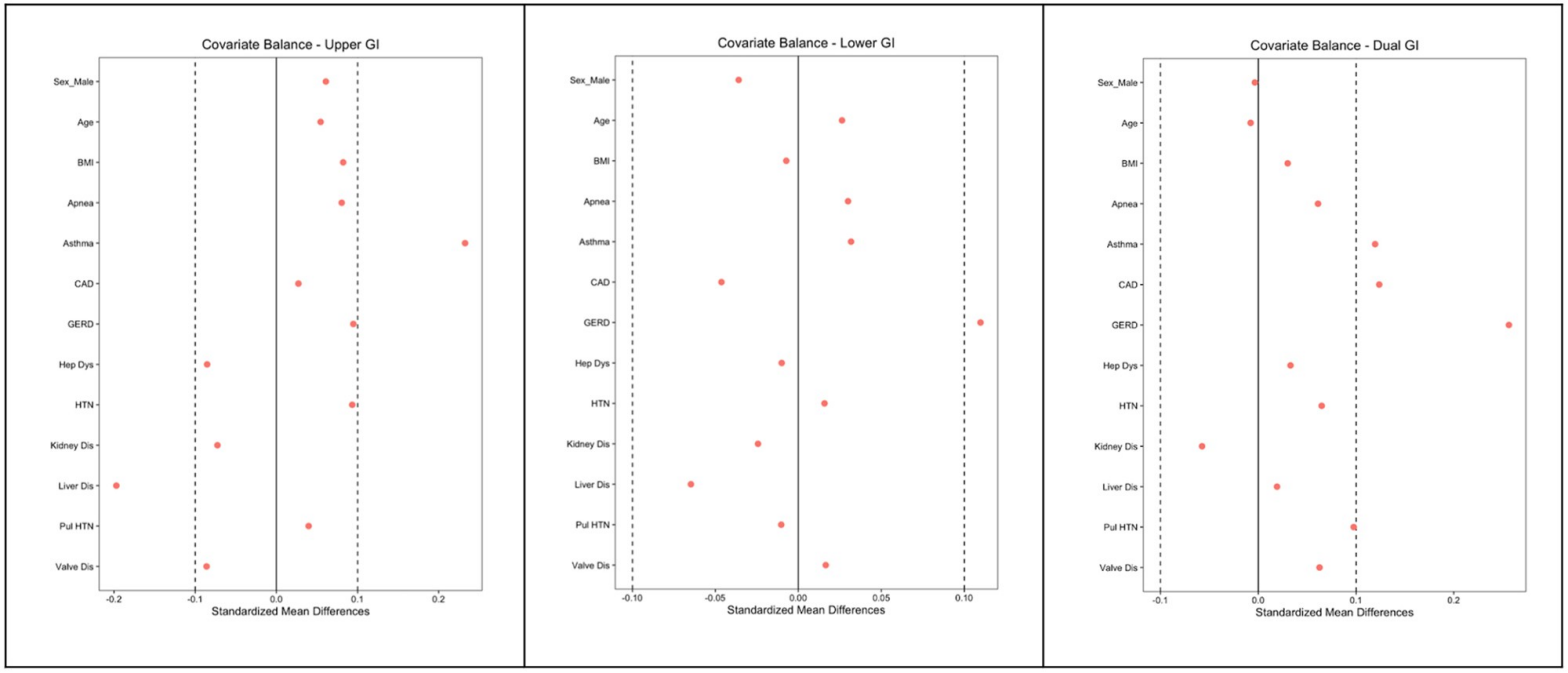
Distribution of clinician satisfaction score, stratified by provider type and sedation type.

A statistically significant difference in satisfaction scores were observed for nurses (MAC average 47.0 points higher than NAS, p = 9.9x10^-10^, 95% CI: 35.9, 58.1) and for proceduralists (MAC average 19.6 higher than NAS, p = 0.0084, 95% CI: 5.5, 33.7). This metric is referred to as the clinician satisfaction score (CSS) (Table 2). Distribution of CSS stratified by provider and sedation type are displayed in Fig 3.

## Discussion

In this retrospective, natural experiment, results demonstrated a statistically significant reduction in the primary outcome of SSSI while using MAC protocols. With MAC protocols, observed reductions in SSSI were attributed to the use of propofol as a sedative, as it has been proven to decrease induction-to-procedure-start time [22], as well as recovery times [23]. Due to propofol’s ability to induce deeper levels of sedation compared to benzodiazepines or opioids [24], administration of the drug demands advanced training should complications arise. Debate remains as to whether RNs or anesthesia professionals can provide equal levels of safe propofol administration during GI procedures, but a consensus deems that specialized training is required to safely administer propofol [25]. As a result, requirements for anesthesia-specific training to administer this sedative is common practice [26], making the use of a physician led anesthesia care team a vital aspect of propofol administration.

The greatest benefit to the improved time values and subsequent increased operational efficiency with MAC protocols, is the ability to increase case volumes and thus enhance patient access through increased procedural room utilization [27, 28]. Process changes that enhance time savings in the procedural room are critical if they allow for additional cases within the same block time. Decreases in SSSI with MAC protocols represent an opportunity to save nearly 12,066 minutes over a 6-month period when applied to the 4,758 NAS cases in this study. Additionally, decreased time attributable to secondary outcomes such as SOOR and PACU LOS—attributed to propofol’s known decreased time-to-awake as compared to benzodiazepines [29]—add secondary avenues for total time savings. Notably, reductions in PACU LOS can be a significant flow improvement for any procedural suite as increasing procedural room utilization can lead to bottlenecks in recovery areas [30].

The secondary operational outcome of in-room to scope-in time (IRSI) further demonstrated significant time reductions to procedural length. It is important to note that IRSI times, while significant both statistically and clinically, were not attributable only to MAC protocols or medication changes but included increased efficiency from process changes. Shifting the consent process out of the procedural rooms resulted in reduced idle time during case times for RNs and anesthesia. Evidence for workflow optimizations improving efficiency have been demonstrated in other healthcare settings [31] and the reductions seen in IRSI are further confirmation that improvements to process flow should be the first consideration for any process improvement initiatives geared toward efficiency.

The projected caseload increases described above are born out in real-time when comparing current GI procedural cases volumes under MAC to previous NAS caseloads at UCH. The initial study compared a six-month period of cases done exclusively with NAS protocols to a comparison group of MAC protocols during a three-month ramp-up period for the new MAC protocols for GI endoscopy. The total six-month case volume during the exclusive NAS period used in this study was 4,758 patients. A post hoc analysis of endoscopic case volumes performed under MAC protocols during the six-month period of 3/1/2023 to 8/31/2023 demonstrated a case volume of 5,838 patients. This represents a 123% increase in patient volume under MAC as compared to the original 6-month NAS group analyzed in this study. This strongly correlated to the change in sedation protocols and consenting process.

It is worth mentioning how the increase in case volume seen in this study could lead to a subsequent increase in procedural suite revenue. Due to this study’s design as a natural experiment and an inability to retrieve financial data secondary to hospital and physician group agreements, this cannot be readily evaluated in the current study. The authors intend on performing a subsequent analysis directed at the assessing the financial impact of these operational efficiencies to confirm the effects on revenue and cost savings.

It was important to ensure that quality of care and clinician satisfaction were maintained in the setting of the changes made during the switch from NAS to MAC. PACU Length of Stay (PACU LOS) and the rate of required PACU pain medication administered post-operatively were used as a proxy measure for the quality of care. Additionally, clinician satisfaction surveys were administered after the implementation of MAC to allow clinicians to rate their relative satisfaction between the two processes. Patients who underwent MAC sedation did not require pain medication while in the PACU which may be attributable to the avoidance of opioid induced nociceptive sensitization [32], a phenomenon that occurs with the administration of fentanyl during NAS protocols. Similarly, PACU LOS times decreased in MAC cohorts as compared to NAS, meaning patients—on average—reached a point of stability that allowed them to leave the PACU at a faster rate than their NAS counterparts. Studies measuring subjective patient satisfaction would need to be explored in future experiments. In terms of clinician satisfaction, MAC protocols showed significant improvements in mean satisfaction scores for both GI proceduralists and nurses. Improved process efficiency and a reduction in post-operative pain are likely the key contributing factors to the improved satisfaction scores. The disadvantages of this approach include the latency between process implementation and survey administration as well as the collection of survey results that are not individualized to their respective processes. However, the advantages of this approach include allowing the clinicians time to familiarize themselves with both processes and make a direct comparison between their experiences to determine their level of satisfaction. Furthermore, given the constraints of this study as a retrospective, natural experiment, there was no opportunity to preemptively administer a satisfaction score for the NAS protocol alone and follow up with a separate MAC survey.

Many of the limitations of this study stem from its design as a natural experiment. One such limitation was deficiencies to data collection. The retrospective nature meant that data not tracked by the EMR was also not able to be followed from the outset of the experiment. Similarly, the change in processes and subsequent type of clinicians administering sedation, led to different types of data being purposefully collected during each process. To mitigate these inconsistencies, outliers representing incorrect time entries were excluded from the analysis, a month-long washout period at the beginning of MAC protocol implementation was allowed for the sedation process to establish a base-line consistency, and a collection period sufficient to gather a large data set for statistically significant analysis was allowed. As nursing staff do not rely on ASA physical classification values, NAS and MAC protocols lacked a standard categorization of patients for sedation. To circumvent this, significant medical comorbidities for both groups were analyzed and stratified across separate identifiers (Table 1) to achieve a similar level of risk assessment and ensure an equal comparison. The ability to track outcomes such as adverse events or to administer patient satisfaction surveys was not possible given these limitations. Instead, proxy measurements that were tracked in the EMR—such as PACU LOS and the rate of PACU pain medication administration—were used to determine pain management and patient stability in the post-operative setting.

The simultaneous manipulation of three independent variables between the two protocols, which included type of sedative, type of clinician providing sedation, and process flow, presented another limitation to this study. Concurrent changes to type of sedative and type of clinician were unavoidable due to the need for advanced training to administer propofol at UCH. However, a discussion is warranted as to which of these variables had a greater impact on outcome improvements. The expertise of the anesthesia care team is a distinguishing component of the MAC sedation protocol. The team-based approach allowed for tighter control and possibly a more rapid achievement of the desired level of sedation while ensuring safe patient care. These reduced physiologic disturbances and faster recovery seen with MAC protocols [33] could be an indication of the enhanced control afforded by the heightened training of the anesthesia care team. However, given the lack of clear evidence regarding the effects of administering clinician on time to sedation, it is more likely the change in sedative class—from benzodiazepines, opioids and antihistamines to propofol—played a greater role in the decreased operational times as has been observed in prior studies [34].

In conclusion, these study results suggest MAC sedation in comparison to NAS significantly improves operational efficiency. These efficiencies represent a distinct opportunity to increase access to care. These efficiencies come at no detriment to the quality of care with decreased need for pain medication during recovery, shorter PACU stays, and increased clinician satisfaction. Finally, these results represent a unique addition to the literature regarding the operational efficiency of sedation and signify a further step toward elucidation of the best practices for sedation during GI procedures.

## Supporting information

S1 File Operational data. Operational data from study period and post hoc analysis of case volumes.
(XLSX)

S2 File Satisfaction scores. GI proceduralist and nursing satisfaction scores.
(XLSX)
